# Resensitization of TRPV1 channels after the P2 receptor activation in sensory neurons of spinal ganglia in rats

**DOI:** 10.3389/fncel.2023.1192780

**Published:** 2023-06-01

**Authors:** Olena A. Petrushenko, Anastasiiya O. Stratiievska, Mariia O. Petrushenko, Elena A. Lukyanetz

**Affiliations:** Department of Biophysics of Ion Channels, Bogomoletz Institute of Physiology, Kyiv, Ukraine

**Keywords:** P2 receptor, TRPV1 channel, capsaicin, ATP, DRG neurons, desensitization, resensitization, calcium ions

## Abstract

**Introduction:**

TRPV1 channels are responsible for detecting noxious stimuli such as heat (>43°C), acid, and capsaicin. P2 receptors are involved in numerous functions of the nervous system, including its modulation and specific response to the application of ATP. In our experiments, we investigated the dynamics of calcium transients in DRG neurons associated with TRPV1 channel desensitization and the effect of activation of P2 receptors on this process.

**Methods:**

We used DRG neurons from rats P7–8 after 1–2 days of culture to measure calcium transients by microfluorescence calcimetry using the fluorescent dye Fura-2 AM.

**Results:**

We have shown that DRG neurons of small (d < 22 μm) and medium (d = 24–35 μm) sizes differ in TRPV1 expression. Thus, TRPV1 channels are mainly present in small nociceptive neurons (59% of the studied neurons). Short-term sequential application of the TRPV1 channel agonist capsaicin (100nM) leads to the desensitization of TRPV1 channels by the type of tachyphylaxis. We identified three types of sensory neurons based on responses to capsaicin: (1) desensitized 37.5%, (2) non-desensitized 34.4%, and (3) insensitive 23.4% to capsaicin. It has also been shown that P2 receptors are present in all types of neurons according to their size. So, the responses to ATP were different in different-sized neurons. Applying ATP (0.1 mM) to the intact cell membrane after the onset of tachyphylaxis caused recovery of calcium transients in response to the addition of capsaicin in these neurons. The amplitude of the capsaicin response after reconstitution with ATP was 161% of the previous minimal calcium transient in response to capsaicin.

**Discussion:**

Significantly, the restoration of the amplitude of calcium transients under the ATP application is not associated with changes in the cytoplasmic pool of ATP because this molecule does not cross the intact cell membrane, thus, our results show the interaction between TRPV1 channels and P2 receptors. It is important to note that the restoration of the amplitude of calcium transients through TRPV1 channels after application of ATP was observed mainly in cells of 1–2 days of cultivation. Thus, the resensitization of capsaicin transients following P2 receptor activation may be associated with the regulation of the sensitivity of sensory neurons.

## 1. Introduction

TRPV1 (transient receptor potential vanilloid 1) channels belong to the receptor-controlled cationic channels of transient receptor potential, which are activated by vanilloids, and provide thermoreception in the range of 33–39°C and nociception. The most well-known activator of TRPV1 receptors is capsaicin (Caterina et al., 1997). Long-term use of capsaicin leads to almost complete desensitization of the TRPV1 channel. An increase in the intracellular concentration of Ca^2+^ ions facilitates this desensitization (Koplas et al., 1997; Mandadi et al., 2004; Liu et al., 2005).

Desensitization of TRPV1 channels to the action of capsaicin can be acute when the receptor loses its ability to respond to the application of an agonist after its long-term application, and tachyphylaxis, which is manifested in a gradual decrease in the maximum amplitude of calcium transients during successive short-term additions of the same concentrations of capsaicin (Petrushenko et al., 2018, 2020). TRPV1 desensitization is, at least in part, a Ca^2+−^dependent process and is reportedly enhanced by dephosphorylation of the receptor by calmodulin-dependent phosphatase 2B (calcineurin; Docherty et al., 1996; Numazaki et al., 2002; Mohapatra and Nau, 2005; Lukacs et al., 2007; Szallasi et al., 2007; Petrushenko et al., 2021).

The process of increased activity (sensitization) of the nociceptor is called hyperalgesia. Sensitization of TRPV1 receptors in the cytoplasm is mediated by various plasma agents (Lopshire and Nicol, 1998; Tominaga et al., 2001; Mandadi et al., 2004; Liu et al., 2005; Petrushenko et al., 2020). On the other hand, some works show the role of the application of agonists for metabotropic receptors on the cell membrane in the sensitization of TRPV1 receptors (Lopshire and Nicol, 1998; Zhang et al., 2005; Petrushenko et al., 2021). The study (Shimizu et al., 2022) shows that intracellular ATP regulates the basal activity of TRPV1 channels in the absence of capsaicin. These results suggest that intracellular ATP functions as a modifier of TRPV1 channel gating.

Application ATP on the plasma membrane activates ionotropic P2X and metabotropic P2Y receptors. This division is based on the molecular structure and mechanism of signal transmission (Roberts et al., 2006; Evans, 2009; Burnstock, 2012; Petrushenko, 2012). ATP, acting on P2 receptors, leads to significant changes in cell membrane permeability (Nicke et al., 1998; Burgard et al., 1999; MacKenzie et al., 1999; Torres et al., 1999).

We hypothesize that activation of P2 receptors has a regulatory effect on TRPV1 channels in rat sensory neurons.

## 2. Methods

### 2.1. Obtaining cultures of DRG neurons

An 8-day-old female rat (P8) was used to obtain a culture of DRG neurons. The rat was sedated with ether, decapitated, and the spinal cord was removed. In total, 15–18 dorsal ganglia were sequentially isolated from the cleaned spinal canal. Freshly isolated cells were placed in a solution of MEM with the addition of HEPES-NaOH (pH 7.4). Ganglion bodies were separated from nerve fibers and placed in a solution containing the enzymes trypsin 2 mM and collagenase 1 mM, which was incubated in a thermostat for 30–35 min at *t* = 37°C. Next, the ganglia were moved into a medium solution containing fetal serum and incubated in a CO_2_ incubator for 10 min to inactivate enzymes. The bodies of the ganglia were freed from the connective tissue sheath, and each was cut into several parts with the help of scissors. After that, 0.5 ml of the medium solution was homogenized with a glass pipette with a hole < 0.5 mm in diameter until the solution became cloudy. A drop of the resulting cell suspension was placed in a Petri dish containing 5–7 glass slides with a diameter of 10 mm and 2 ml of medium solution. After that, the glass with the neuron suspension was kept in a CO_2_ incubator for 24 h to 7 days, with periodic replacement of the culture medium every 3–5 days.

### 2.2. Experimental solutions and media for cell cultivation

The isolation solution is aqueous on MEM with (mM) 10HEPES and a pH of 7.4.

The culture medium containing 90% DMEM liquid medium and 10% inactivated fetal calf serum, 2 mM NaHCO_3_, 2 mM glutamine, 5 mM sodium pyruvate, 0.6 μM dry bovine insulin, and a penicillin/streptomycin mixture (0.03%) was added to the resulting solution.

The base solution for the experiments is an aqueous Tyrode solution containing (in mmol/l) 125 NaCl; 2.5 KCl; 2 CaCl_2_, 1 MgCl_2_, 10 glucose, and 20 HEPES. The required concentration of CaCl_2_ (1–3 mM) was added under the experimental conditions. The pH of the solution was adjusted to 7.35–7.40.

Extracellular solution with an increased concentration of KCl contained (in mmol/l) 78 NaCl; 50 KCl; 2 CaCl_2_, 1 MgCl_2_, 10 glucose, and 20 HEPES. The pH of the solution was adjusted to 7.35–7.40.

The ATP and capsaicin solutions used in the experiments were prepared based on Tyrode's basic solution and contained 100 μM ATP and 1 μM capsaicin, respectively.

All reagents used to prepare the above solutions were purchased from Sigma, USA.

### 2.3. Measurement of intracellular Ca^2+^ level (calcium imaging)

Intracellular calcium concentration was measured using an experimental setup consisting of an optical microscope (Olympus IX70 Tokyo, Japan), an experimental camera fixed on the microscope platform, image recording and data logging software (Cell M Imaging Software, Olympus, Tokyo, Japan), local application system, a monochromator, and a light source (Illumination System MT10, Olympus, Tokyo, Japan).

To measure the intracellular concentration of calcium ([Ca^2+^]i), we used the common calcium-sensitive dye Fura-2, which was loaded into the DRG cells using the lipophilic form of the Fura-2 AM dye (Molecular Probes, USA), which can penetrate the cell membrane. After Fura-2 AM enters the cell, the dye is cleaved by intracellular esterases and converted into a hydrophilic, calcium-sensitive form (Fura-2), which no longer penetrates the cell membrane.

Freshly isolated DRG neurons were incubated for 30 min at 37°C in a base solution containing 1–2 μm Fura-2 AM. After that, the cells were washed with a pure basic solution and left in the dark at 37°C for 30–40 min for the final de-esterification of Fura-2 AM. A Petri dish with glass on the surface of which the cells were attached was placed in the field of view of an Olympus IX70 inverted microscope (Tokyo, Japan) with a short-focus objective with a magnification of x20. The measurement and data recording processes were managed using the Cell M Imaging Software (Olympus, Tokyo, Japan). A local application system changed the extracellular solution around the selected cell. The experimental setup allowed working in two modes. Thus, the object was illuminated in the setting mode by a visible light source. In registration mode, the installation was set to illuminate the object using a xenon lamp. In this mode, the fluorescent probe is excited at 340 and 380 nm wavelengths using appropriate filters. The emission of the probe was recorded in the ranges of 395–415 nm (F1) and 470–490 nm (F2) using two photomultipliers. The signals from the photomultipliers were fed to the input (DIV100, Burr Broun, USA), which in real time calculated the ratio of fluorescent probe signals at two wavelengths of excitation light: Ratio = F1/F2 = F340/F380. The obtained experimental data were digitized and stored on the computer's hard disk for further processing.

### 2.4. Statistical processing of the obtained results

The analysis of experimental data was carried out using the program Microcal^TM^ Origin Pro^TM^, version 8.5 (Microcal Software Inc., USA). Based on the obtained values, histograms of the distribution of the “Microcal^TM^ Origin Pro^TM^” program, version 8.5, were constructed. Finding the most probable values was carried out by the standard method of approximating the obtained histograms with the Gaussian distribution. Data groups were assessed for normality using the “Origin Pro^TM^” software as the Shapiro–Wilk normality test. Statistical hypotheses were assessed using the Student's *t*-test.

## 3. Results

### 3.1. The role of purinoreceptors in generating ATP-induced calcium transients in DRG cells

Our studies by the method of microfluorescence calcimetry showed that the sequential injection of ATP (5 s) with an interval of 60 s into neurons of the dorsal ganglion causes a rapid and short-term increase in the concentration of cytosolic calcium [Ca^2+^]i. The concentration of [Ca^2+^]i reaches a maximum of 2–4 s after 3–5 s of ATP application and returns to the basal level after ATP was removed from the solution.

To study the dynamics of calcium entry into neurons, we applied ATP several times with an interval of 60 s.

The first type of response developed if the successive application of ATP caused a gradual decrease in the amplitude of Ca^2+^ transients (by 30 ± 4%) compared to the first (control) application. Continuation of such applications every 20–25 s led to a decrease in the amplitude of transients almost to zero in some neurons. This decrease occurred as a result of the desensitization of Ca transients by the type of tachyphylaxis.

The second type of response was characterized by the preservation of the amplitude of calcium transients in the conditions of several consecutive applications of ATP and the non-availability of desensitization of P2 receptors.

Our results show that P2 receptors are expressed in DRG neurons of different diameters. In most cells of medium and large diameters, consecutive applications of ATP with an interval of 60 s did not cause a noticeable decrease in the amplitude of [Ca^2+^]i transients. Thus, we conclude that P2 receptors that do not show desensitization are predominantly present in most medium- and large-diameter cells.

**Figure 1:**
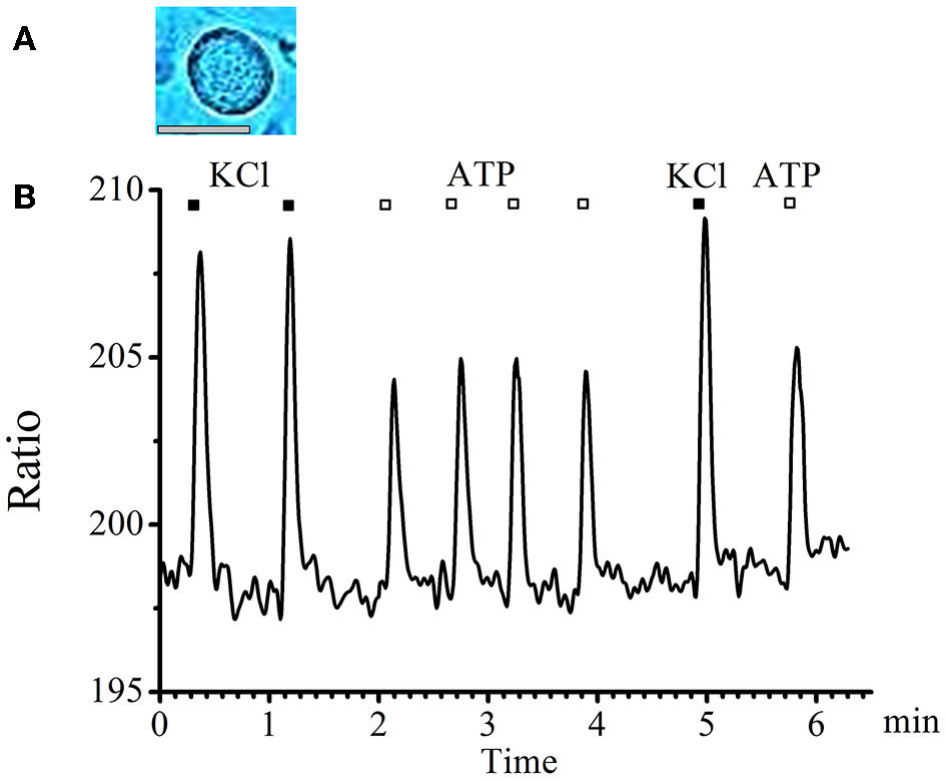
Absence of tachyphylaxis of calcium transients in response to ATP applications in large- and medium-diameter neurons. **(A)** Image of a typical neuron with an medium diameter (scale bar 25 μM long). **(B)** Recording of calcium transients in medium-diameter cells in response to the standard depolarizing agent −50mM KCl (black rectangles) and to the tested substance −0.1 mM ATP (white rectangles).

A typical graph of calcium transients in cells of medium and large diameters in response to a standard depolarizing agent—KCl and to the studied substance—ATP is presented in Figure 1.

**Figure 2:**
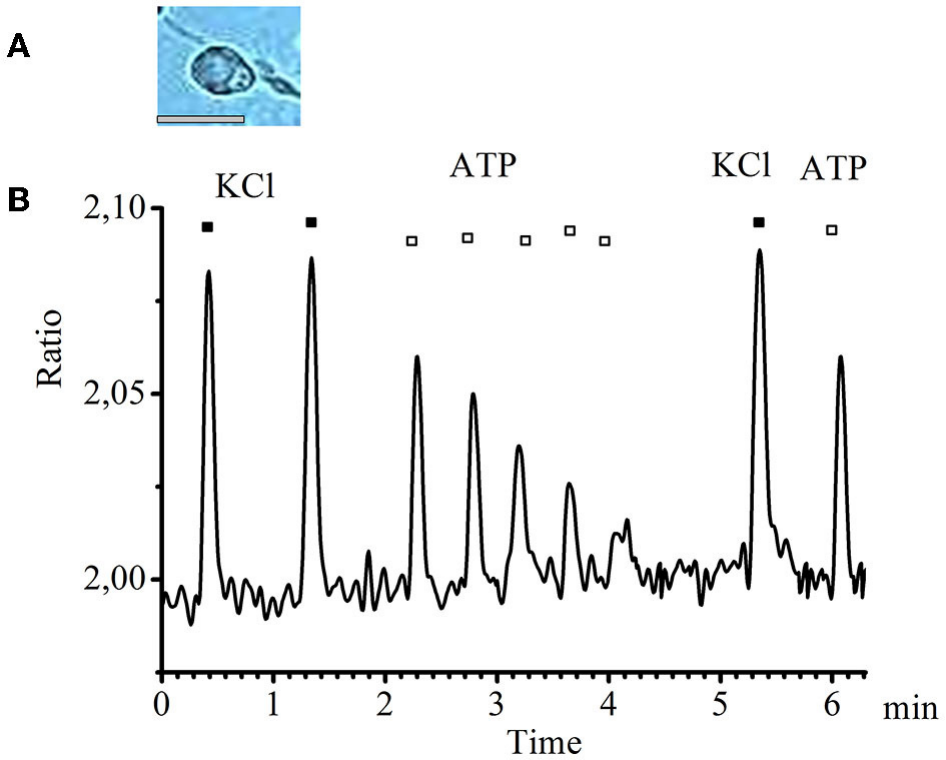
Desensitization of calcium transients in response to ATP application in small-diameter neurons. **(A)** Image of a typical neuron with a small diameter (scale bar 25 μM long). **(B)** Recording of calcium transients in cells of small diameter in response to the standard depolarizing agent −50 mM KCl (black rectangles) and to the investigated substance −0.1 mM ATP (white rectangles).

In small-diameter cells, a decrease in the amplitude of transients was observed in response to ATP applications with an interval of 20–25 s starting from the second application until the transients completely disappeared. A typical graph of such transients obtained experimentally is presented in Figure 2.

Thus, we conclude that P2 receptors that show clear desensitization are present in the small-diameter cells.

### 3.2. Investigation of capsaicin-induced calcium transients through TRPV1 channels in the membrane of DRG neurons

Cultivated cells of DRG ganglia used in the experiment showed a relationship between the activity of neurons in response to the application of an agonist and their size. We measured the largest cell diameter in linear dimensions. Small neurons were considered d < 22 μm (8% of total cells), medium-sized neurons had d = 24–32 μm (22%), and large neurons had d = 32–48 μm (63%).

Desensitization of TRPV1 channels is called acute when the receptor loses its ability to respond to the application of an agonist after its long-term application, and tachyphylaxis, which is manifested in a gradual decrease in the maximum amplitude of calcium transients during successive short-term additions of the same concentrations of capsaicin (Koplas et al., 1997; Liu et al., 2005). As it was shown earlier, there is a specific heterogeneity of the studied DRG cells in terms of the dynamics of activation and desensitization of calcium transients of TRPV1 channels under the action of capsaicin (Petrushenko et al., 2020). This study also identified three types of sensory neurons maintained in a primary culture based on responses to capsaicin: (1) desensitized 37.5%, (2) non-desensitized 34.4%, and (3) insensitive 23.4%. When applying capsaicin (after 2 min), desensitization of TRPV1 channels was observed in 37.5% of neurons, which lasted until the complete cessation of calcium entry.

**Figure 3:**
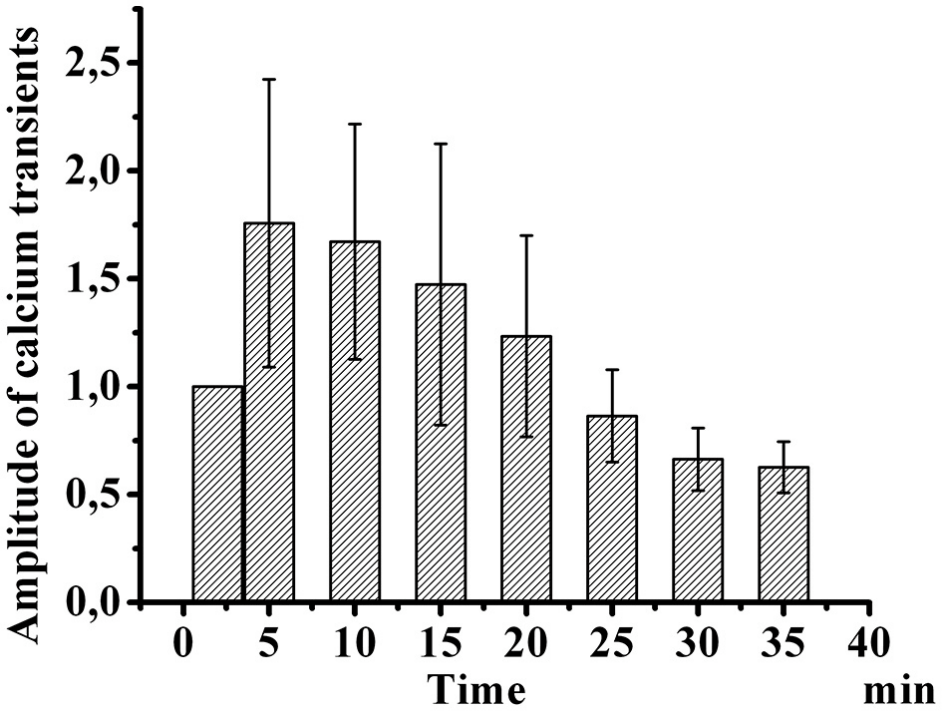
Desensitization of calcium transients in response to capsaicin application in small-diameter neurons. The diagram shows changes in the amplitude of successive short-term applications of capsaicin over time (100 nM, 5 s). The y-axis illustrates normalized averaged amplitudes of calcium transients in response to capsaicin application. Means and S.E. shown data groups were assessed for normality using the software “Origin Pro^TM^” as the Shapiro–Wilk normality test.

Figure 3 shows the development of desensitization of TRPV1 channels by the type of tachyphylaxis. Successive short-term application of Caps (100 nM, 5 s) to the neuron membrane with a 2-min washout after each application activated TRPV1 channels and led to their desensitization. As shown in Figure 3, repeated use of Caps at a low concentration leads to a clear desensitization of TRPV1 channels, manifested in a gradual decrease in the maximum amplitude of calcium transients.

### 3.3. Interaction between TRPV1 and ATP receptors in DRG neurons

**Figure 4:**
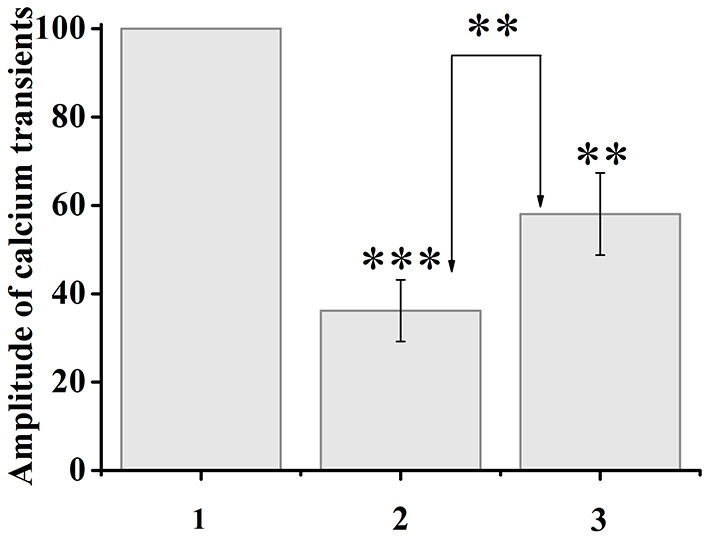
Application of ATP (100 μM, 20 s) to capsaicin-desensitized neurons caused resensitization of TRPV1 channels. The y-axis illustrates normalized to the maximal peak averaged amplitudes of calcium transients in response to capsaicin application. (1) Maximal peak at the start of the experiment, (2) minimum amplitude during desensitization, (3) resensitization of calcium transients after ATP application. The numbers in the second and third columns of the chart indicate the percentage increase. Data sets were evaluated for normality by the Shapiro–Wilk normality test. Statistical hypotheses were evaluated by Student's *t*-test. ***p ≤ 0.001 (2) vs. (2) between desensitization transients; **p ≤ 0.01 (2) vs. (3) between desensitization transients and resensitization transients; **p < 0.01 (3) vs. (3) between resensitization transients.

Capsaicin activates ligand-gated TRPV1 channels, which leads to an increase in the concentration of intracellular ionized calcium. When capsaicin was added (after 2 min), desensitization of TRPV1 channels was observed in 37.5% of neurons by the type of tachyphylaxis until the complete cessation of calcium entry. At the same time, the addition of ATP (100 μM, 10–20 s) 5 min after the onset of desensitization, immediately before the next capsaicin application, caused an increase in calcium transients in response to capsaicin application in 35.7% of desensitized neurons. The calcium transient amplitude after sequential application of ATP and capsaicin was 161% of the previous minimal calcium transient in response to capsaicin of the same neuron. A comparison of the normalized values of the amplitudes of the responses to capsaicin before and after the application of ATP is shown in Figure 4.

**Figure 5:**
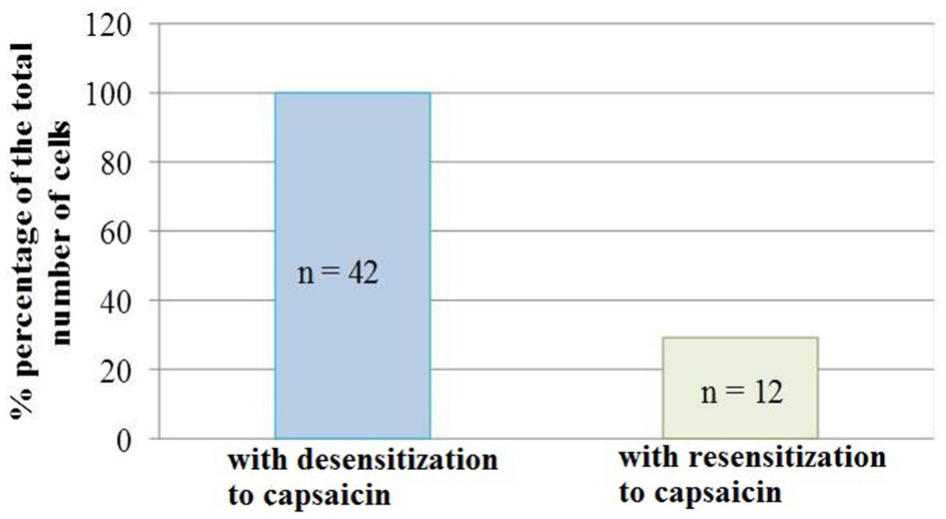
Diagram showing the ratio of cell number which demonstrated the desensitization to capsaicin (100%, *n* = 42) and resensitization (29%, *n* = 12) of calcium transients in conditions of co-application of capsaicin and ATP. Data sets were evaluated for normality by the Shapiro–Wilk normality test.

It should be noted that not all neurons showed resensitization of calcium transients in response to activation of P2 receptors. The number of cells that showed an increase in calcium transients under conditions of simultaneous application of ATP and capsaicin was 29% of the total number of cells that showed desensitization to the action of capsaicin (Figure 5).

## 4. Discussion

TRPV1 and purine receptors play a significant role in nerve cell activity. Interaction between the two has been observed in a variety of processes, including pain sensation, synaptic plasticity, and synaptic transmission (Yu et al., 2021). TRPV1 is a non-selective cation channel that is found throughout the central and peripheral nervous systems. It has been linked to a variety of processes and has been widely studied due to its involvement in pain sensation. Activation of TRPV1 receptors can lead to the release of a variety of neurotransmitters, such as calcitonin gene-related peptide (CGRP) and substance P.

Regulation of TRPV1 channels can also occur through modulation by intracellular molecules such as Ca^2+^ ions (Koplas et al., 1997), phosphatidylinositol 4,5 bisphosphate (PIP2; Prescott and Julius, 2003), or calmodulin (Rosenbaum et al., 2004). TRPV1 channels are inactivated upon binding to PIP2 and re-sensitized through PKC-mediated hydrolysis of PIP2 (Prescott and Julius, 2003). Due to the activation of protein kinase C (PKC) and phosphorylation of TRPV1 channel proteins, the channels can participate in interaction with other receptors, for example, P2Y1 (Tominaga et al., 2001) or P2Y2 (Lakshmi and Joshi, 2005; Wang et al., 2010). Malin et al. (2008) showed that P2Y2 receptors found in sensory neurons can be co-expressed with TRPV1 receptors.

Purine receptors are another important player in nerve cell activity. They are involved in synaptic plasticity, the ability of neurons to change their response to a stimulus (Ren and Bertrand, 2008). Purine signaling has been linked to the release of neurotransmitters, such as glutamate and GABA, and has been shown to modulate synaptic transmission.

The interaction between TRPV1 and purine receptors has been observed in many processes (Tominaga and Moriyama, 2007). In pain sensation and synaptic plasticity, TRPV1 activation leads to the release of CGRP, which can then activate purine receptors, leading to the release of glutamate and GABA.

ATP activation of P2Y2 receptors leads to activation of Gq-proteins of the mediated metabolic pathway (Erb and Weisman, 2012) with subsequent phosphorylation of channel proteins and resensitization of TRPV1 channels.

Overall, the interaction between TRPV1 and purine receptors is essential for normal nerve cell activity.

In the study (Khmyz et al., 2008), it was found that the P2X3 receptor is responsible for temperature sensitivity. It is shown that the development of desensitization of the P2X3 receptor does not depend on the temperature in the range of 25.40°C; at the same time, recovery after desensitization is significantly accelerated with increasing temperature. The authors conclude that the unusual combination of sensitivity/insensitivity of P2X3 receptors may be related to the key role of these receptors in thermosensitivity. It should be noted that TRPV1 channels are also associated with hot temperature perception (>43°C). Thus, resensitization of TRPV1 channels after P2X receptor activation may be associated with increased sensitivity regulation.

In the work (Glaser et al., 2013), the authors conclude the participation of purinergic receptors in controlling metabolic activities and many physiological functions but in different ways: while most P2Y receptors induce transient elevations of intracellular calcium as waves, P2X receptors produce fast calcium spikes.

Among DRG neurons, nociceptive C-fiber neurons belong to a subset of small cells. In small sensory neurons of rats, two populations of nociceptors were isolated – functional homomeric P2X3 and heteromeric P2X2/3 receptors (Nicke et al., 1998; MacKenzie et al., 1999; Torres et al., 1999). The time of the open state of the ion channel depends on the subunit composition of the receptors. For example, P2X3 receptor channels desensitize rapidly in the presence of ATP (several hundred milliseconds), while the P2X2 receptor channel remains open as long as ATP is associated with it (Torres et al., 1999). Another study showed that ATP elicits P2X receptor-mediated responses in rat DRG neurons with different desensitization kinetics - slow non-desensitizing kinetics, fast desensitizing kinetics, or a combination of fast and slow kinetics. The authors conclude that rapid desensitization responses were similar to P2X3 receptor responses, and responses without desensitization had properties indicative of P2X2 or P2X2/3 receptor activation (MacKenzie et al., 1999).

In our experiments, the majority of medium- and large-diameter cells did not show a significant decrease in the amplitude of calcium transients through P2 receptors after successive applications of ATP. Thus, we concluded that P2 receptors, which do not show desensitization, are present mainly in cells of medium and large diameters. In small-diameter DRG neurons, observed calcium transitions through P2 receptors in response to ATP application demonstrate pronounced desensitization. Thus, P2 receptors with desensitization are expressed in these neurons.

It should be noted that when comparing the responses of the studied cells on days 1-2 and 5-7 of cultivation, differences were found in the number of each of the groups of cells in terms of sensitivity to capsaicin. On the 5-7th days of cultivation, changes occur in neurons associated with their adaptation to the conditions of cultivation. Accordingly, there is a significant reduction in the number of cells that respond to capsaicin. It is important to note that the restoration of the amplitude of calcium transients under the influence of ATP was observed mainly in cells on the 1st to 2nd days of cultivation. Thus, under the conditions of a short cultivation time, neurons retain the properties of freshly isolated cells and there are no transformations associated with adaptation to cultivation conditions.

In conclusion, we found that when DRG neurons are desensitized to capsaicin, activation of P2 purinoceptors can restore calcium transients. The amplitude of calcium transients in response to capsaicin after the application of ATP was 161% of the desensitized calcium transient in response to capsaicin.

It is known that the activation of P2 receptors under the influence of ATP is an important factor affecting the formation of inflammation or visceral pain (Chizh and Illes, 2000). Thus, we have shown that ATP-induced resensitization of TRPV1 channels makes them possible participants in elevated calcium responses and the development of these disease states.

## Data availability statement

The original contributions presented in the study are included in the article/supplementary material, further inquiries can be directed to the corresponding author.

## Ethics statement

The animal study was reviewed and approved by Committee for Biomedical Ethics.

## Author contributions

All authors listed have made a substantial, direct, and intellectual contribution to the work and approved it for publication.
